# Methods of staining and visualization of sphingolipid enriched and non-enriched plasma membrane regions of *Arabidopsis thaliana* with fluorescent dyes and lipid analogues

**DOI:** 10.1186/1746-4811-8-28

**Published:** 2012-08-06

**Authors:** Jörg O Blachutzik, Fatih Demir, Ines Kreuzer, Rainer Hedrich, Gregory S Harms

**Affiliations:** 1 Institute for Molecular Plant Physiology and Biophysics, University Würzburg, Julius-von-Sachs Platz 2, D-97082, Würzburg, Germany; 2 Microscopy Group, Rudolf Virchow Center, University of Würzburg, Josef Schneider Str. 2, D15, D-97080, Würzburg, Germany; 3 Present address: Institute of Neuro- and Sensory Physiology, Düsseldorf University Hospital, Universitätsstr. 1, D-40225, Düsseldorf, Germany; 4 Departments of Biology and Physics, Wilkes University, 84 W. South St., Wilkes-Barre, PA 18766, USA

**Keywords:** Protoplasts, Lipid polarization, Lipophilic fluorescent dyes, Laurdan, Sphingolipid, Liquid (dis-) ordered phase, Plasma membrane, Fluorescence microscopy

## Abstract

**Background:**

Sterols and Sphingolipids form lipid clusters in the plasma membranes of cell types throughout the animal and plant kingdoms. These lipid domains provide a medium for protein signaling complexes at the plasma membrane and are also observed to be principal regions of membrane contact at the inception of infection. We visualized different specific fluorescent lipophilic stains of the both sphingolipid enriched and non-sphingolipid enriched regions in the plasma membranes of live protoplasts of *Arabidopsis thaliana*.

**Results:**

Lipid staining protocols for several fluorescent lipid analogues in plants are presented. The most emphasis was placed on successful protocols for the single and dual staining of sphingolipid enriched regions and exclusion of sphingolipid enriched regions on the plasma membrane of *Arabidopsis thaliana* protoplasts. A secondary focus was placed to ensure that these staining protocols presented still maintain cell viability. Furthermore, the protocols were successfully tested with the spectrally sensitive dye Laurdan.

**Conclusion:**

Almost all existing staining procedures of the plasma membrane with fluorescent lipid analogues are specified for animal cells and tissues. In order to develop lipid staining protocols for plants, procedures were established with critical steps for the plasma membrane staining of Arabidopsis leaf tissue and protoplasts. The success of the plasma membrane staining protocols was additionally verified by measurements of lipid dynamics by the fluorescence recovery after photobleaching technique and by the observation of new phenomena such as time dependent lipid polarization events in living protoplasts, for which a putative physiological relevance is suggested.

## Background

Lipid domains in the plasma membrane (PM) help to cluster proteins in eukaryotic cells. They serve as transient domains for the attachment of proteins mainly involved in signal transduction, especially from cells from the animal kingdom [1]. More recently, much evidence has accumulated to indicate that membrane domains play important roles in the defense against pathogens and also in the cell signaling in plants [2]. Lipid domains, also called “lipid rafts”, in the plasma membrane are spatially organized platforms structurally defined by an enrichment of sterols and sphingolipids [3,4]. From a historical perspective, individual lipid components within membrane domains are biochemically associated according to their resistance to treatment with mild non-ionic detergents such as Triton X-100 [5] and are commonly referred to as detergent-resistant membranes (DRMs). Detergent treatment leads to artifacts, and detergent resistance does not necessarily imply lipid rafts [6,7]. However, the enrichment of a specific protein in DRMs suggests an affinity for a distinct lipid environment and indicates localization in membrane subfractions that correlate to lipid nanodomains [8], but DRMs are purely biochemical in nature and do not allow one to specifically observe the domains on the plasma membrane. It is now suggested that only through the direct observation of the domains on the plasma membrane by microscopy one can truly make the claim of “lipid raft” [9]. Lipid rafts have primarily been characterized in the animal kingdom as nanodomains in the plasma membrane where they serve as signalling platforms [1]. Following the concept that these sterol- and sphingolipid-enriched structures are highly dynamic in position and composition it is assumed that individual nanodomains can be stimulated to coalesce into larger, more stable domains by specific lipid-lipid, lipid-protein and protein-protein oligomerizing interactions [10]. In native plasma membranes of plants and fungi similar structures of high lipid order seem to exist, leading towards a lateral segregation of membrane components [8,11]. In plants and fungi plasma membrane domains appear to be quite stable in location, exhibiting less lateral mobility in the bilayer than the lipid raft-counterpart in animals; therefore, these structures should be referred to as membrane (micro-) domains to better distinguish between membrane domains in animals and plants/fungi [9]. Especially in fungi there are striking hints that point towards the coexistence of different membrane compartments of individual protein composition [11,12].

The observation of lipid raft domains in animal cells through fluorescent lipid analogues has produced key results towards the essential knowledge that i) the domains are of the liquid ordered phase whereas the non-domain regions tend to be in the liquid disordered phase, ii) the domains have distinctly smaller sizes and relatively lower mobility than the non-domain regions and iii) the fluorescent lipid analogues specific to the domains tend to specifically appear in the DRM fraction, creating a link from biochemical to microscopic observation.

Among the proteins found in DRM fractions isolated from eukaryotic plasma membranes GPI-anchored proteins as well as subsets of membrane attached proteins involved in signaling were enriched, implicating a physiological importance for the clustering of sphingolipids and sterols [7,13,14]. These lipid assemblies seem to be essential for eukaryotic systems. In vital eukaryotic nerve cells sterol depletion experiments resulted in a depletion of ligand-dependent activation of receptor tyrosine kinases at the plasma membrane ( [15] and references therein). In yeast it was shown that several membrane subcompartments exist in which proton-coupled amino acid/nucleotide/sugar transporters, sphingolipids and sterols accumulated [12]. In plants membrane patches could be visualized by expressing a fluorescently tagged Remorin protein from potato that showed a strong sterol-dependency *in vitro*[2,16]. The biochemical identification of lipid and protein components in plasma membrane domains has been investigated particularly in mammalian cell types and progressively in yeast but yet inert in plants [9,17]. Despite the experimental evidence in the plant kingdom that sterol-dependent proteins exist, there have been, to our knowledge, no efforts been made to selectively label liquid (dis-) ordered membrane compartments in viable plant cells using combinations of fluorescent dyes and lipid analogues. This might have to do with the fact that most of the commercially available dyes suited for confocal microscopy are specifically designed to stain the plasma membranes of animals and not have been reliably shown for plants.

Like that of animals, the plant plasma membrane is laterally segregated. Membrane domains show a lower lateral mobility than their lipid raft-counterparts in animals but also differ regarding the lipid composition, most notably with respect to the complex sterol profile. In Arabidopsis the most abundant sterols are cholesterol, sitosterol, stigmasterol and campesterol, while cholesterol is the predominating sterol in vertebrate plasma membranes [18]. The overall lipid composition in Arabidopsis consists of 37.7 mol% sterols, 15.6 mol% glycolipids (among them 6.8 mol% glycosphingolipids) and 46.7 mol% phospholipids [19]. More recently it was estimated that the content of sphingolipids within the plasma membrane of plants could make up to > 40% [20]. Due to their complex sterol profile plant plasma membranes might be less sensitive towards thermal shocks compared to animal systems [18].

Hence, if physiological plant plasma membranes are subdivided into sphingolipid enriched and non-sphingolipid enriched regions, a clearly different picture from the animal cell view might emerge. Individual lipid domains might show a wider variety of sterol-/sphingolipid assemblies - due to the complex sterol profile of plants. However, it could also be conceivable that the whole plant plasma membrane might function as a liquid ordered compartment at macro scale.

From experiments using model membranes there is proof that different lipid phases can coexist, depending on the lipid composition of the system and on temperature [21,22]. At low temperatures lipid mixtures consisting of solely glycerophospholipids and sphingolipids appear to exist as gel-phase (L_β_); in the L_β_-phase the hydrophobic lipid tails are in the *all-trans* position fully expanded and are accordingly strong and lipids are densely packed. As a consequence lipids resident in L_β_-phases hardly can move. With increasing temperature glycerophospholipids and sphingolipids show a phase transition and appear to exist as fluid-crystalline phase (L_α_). In the L_α_-phase attractive Van-der-Waals forces are decreasing and lipids exhibit a higher degree of lateral movement [23,24]. Phase transitions induce changes in the order of the system. For each lipid species the phase transition temperature is defined [25]. If cholesterol is added to lipid mixtures consisting of glycerophospholipids and sphingolipids, another phase is forming, that reflects an intermediate between L_α_- and L_β_-phases. This phase is called liquid ordered (L_o_)-phase [24]. At the same time the loosely packed lipids of the L_α_-Phase form the liquid disordered (L_d_)-phase [26]. In ternary lipid mixtures consisting of phosphatidylcholin, sphingomyelin and cholesterol L_o_-phases were enriched in sphingomyelin and cholesterol, while L_d_-phases mainly consisted of phosphatidylcholin [27,28]. Sterols enhanced the existence of L_o_-phases since they lower the enthalpy of the L_β_/L_α_-phase transition. These differences in enthalpy disappeared at a cholesterol content of 50 mol% [26]. Nevertheless, the basic mechanisms enabling lipid phase transitions are not yet fully understood [29,30].

Here, different lipid compartments in the plasma membranes of living plant tissues and cells have been visualized using one- and two-photon microscopy. Beyond establishing simple and successful staining protocols for FM4-64, LRB-PE, DiIC_12_, DiIC_18_, DiD, Bodipy-labeled C_12_ Sphingomyelin (BD-SM) and Laurdan for plant cells, we observed evidence for the existence of different lipid phases in protoplasts that are reminiscent of lipid ordered/lipid disordered phases in model membranes. These findings were strengthened by accompanying lipid dynamics of the individual lipid phases and by means of the relative degree of lipid order, as documented by Laurdan. Laurdan imaging demanded an employment of two-photon microscopy to excite fluorescent naphthalene moieties.

## Results

**Figure 1:**
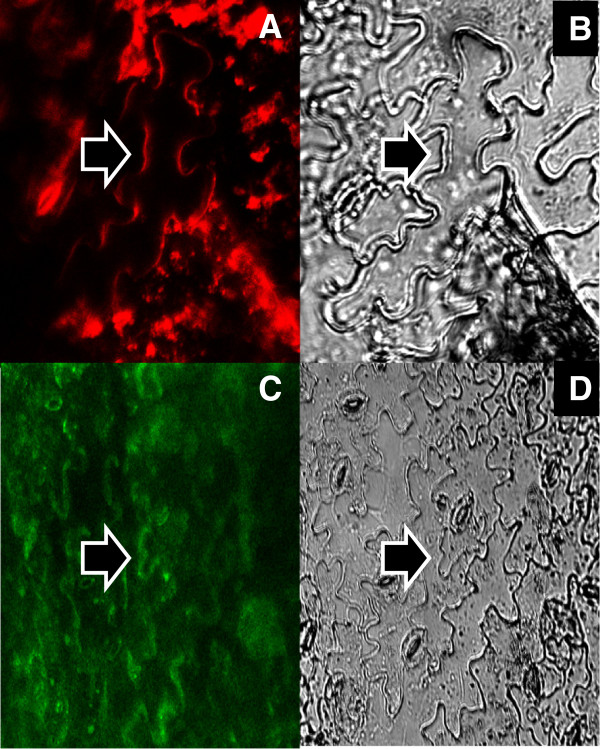
**Whole tissue staining of *A. thaliana *epidermal strips.** Whole tissue staining of *A. thaliana* epidermal strips, using FM4-64 and BD-SM; (**A**) FM4-64 stain; according to the white light image (**B**) some of the fluorescent structures imaged were of the plasma membrane (arrows); (**C**) BD-SM stain; compared to the white light image (**D**) a successful plasma membrane staining was achieved (arrows). Nevertheless, strong background fluorescence signals did not allow for proper imaging using epidermal strips.

**Figure 2:**
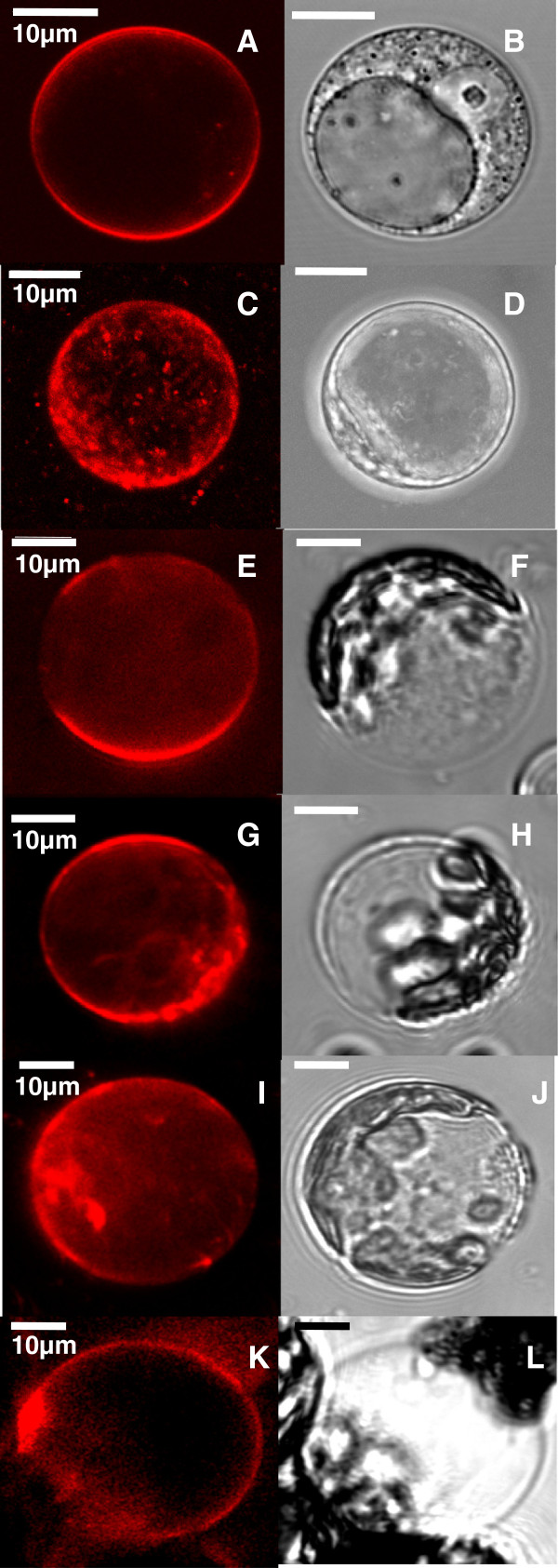
**Single dye staining of protoplasts.** Single dye staining of protoplasts, using FM4-64 (**A**; **C**), LRB-PE (**E**), DiIC_12_ (**G**), DiIC_18_ (**I**), and DiD (**K**); all dyes appeared at the plasma membrane. Upon FM4-64 treatment first fluorescent vesicles appeared in the cytoplasm within 20 minutes; endocytosis events were strongly enhanced with ongoing incubation time (**C**; 30 minutes post FM4-64 incubation). All staining protocols were designed with respect to cell viability; macroscopically the protoplasts were not affected and maintained their round shape (**B**; **D**; **F**; **H**; **J**; **L**). Viability was successfully tested using trypan blue.

**Figure 3:**
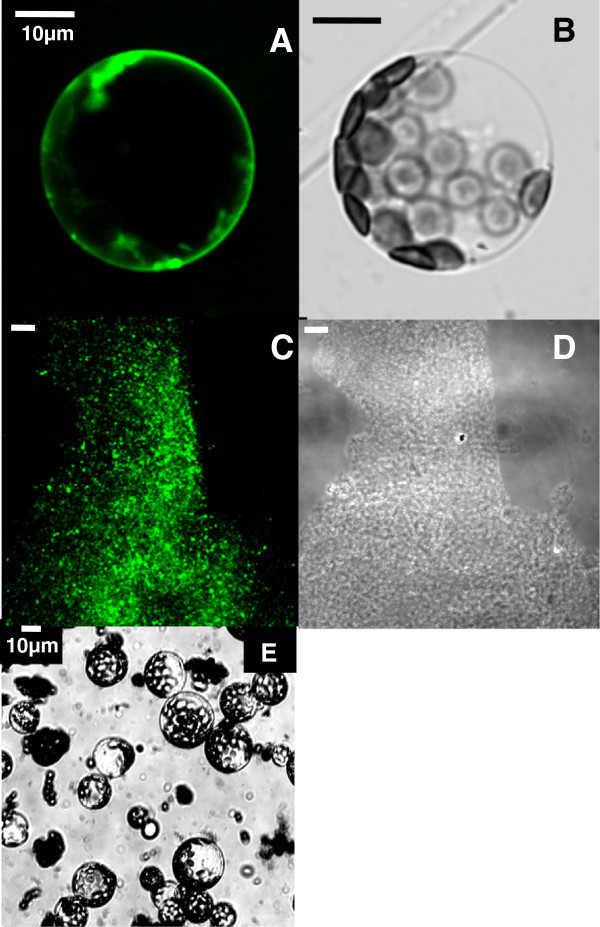
**BD-SM at the plasma membrane and in the DRM-fraction.** BD-SM appearance at the plasma membrane and in the DRM-fraction. (**A** &**B**) BD-SM reliably stained plasma membranes in protoplasts without losing cell viability. Fluorescence (**A**) and transmission image of (**B**) a BD-SM stained protoplast. BD-SM fluorescence was specific to the plane of the membrane (**C** &**D**). When applied to purified plasma membranes BD-SM appeared in the Arabidopsis DRM-fraction. Fluorescence (**C**) and transmission image (**D**) of purified membranes from the DRM-fraction. (**E**) is showing a bunch of protoplasts after trypan blue treatment; trypan blue is excluded from the cytosol of intact cells (here shown after FM4-64 treatment; in protoplasts treated with LRB-PE, DiIC_12_, DiIC_18_, DiD, BD-SM and Laurdan similar results were obtained using trypan blue).

**Figure 4:**
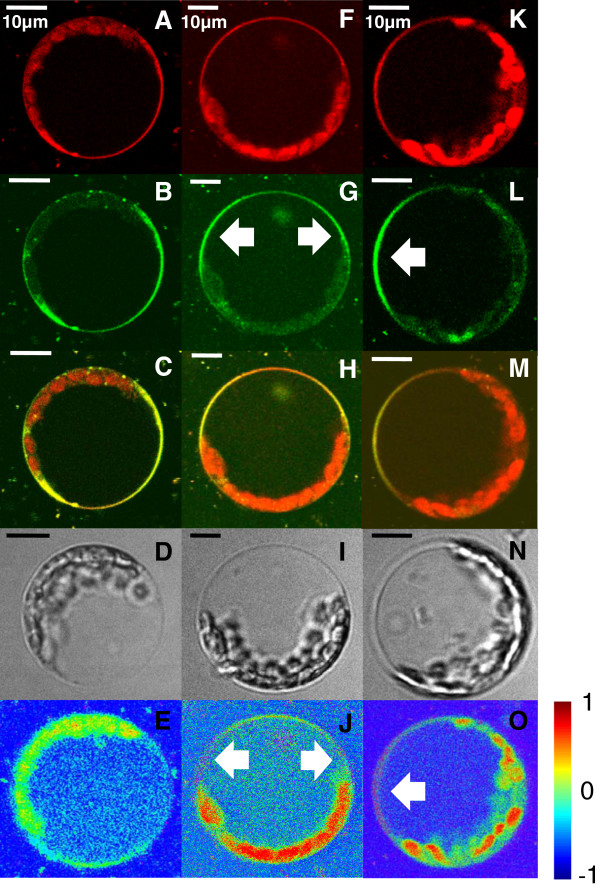
**Time-dependent polarization of protoplasts with Laurdan.** Time-dependent polarization of protoplasts as indicated by Laurdan; the more red shifted emission spectrum of Laurdan (**A**; **F**; **K**) indicated polar phases of higher water content, whereas a more blue shifted emission spectrum (here: green, false color; **B**; **G**; **L**) indicated apolar phases harboring less water; (**C**; **H**; **M**): merged images; (**E**; **J**; **O**): GP-values, corresponding to the GP-scale (−1: pure water phase; +1: fully ordered phase). (**A**-**D**) in freshly isolated protoplasts there was no polarization detectable, GP-values ranged from −0.3 to 0.2 (**E**); (**F**-**J**) 15 h post cell wall removal a lipid polarization was detected; at the lateral sides of the membrane areas of high lipid order emerged (**J**, arrows); (**K**-**N**) 20 h post cell wall removal a wide part of the plasma membrane appeared as polarized (**O**, arrow), accompanied by GP-values of up to 0.8.

**Figure 5:**
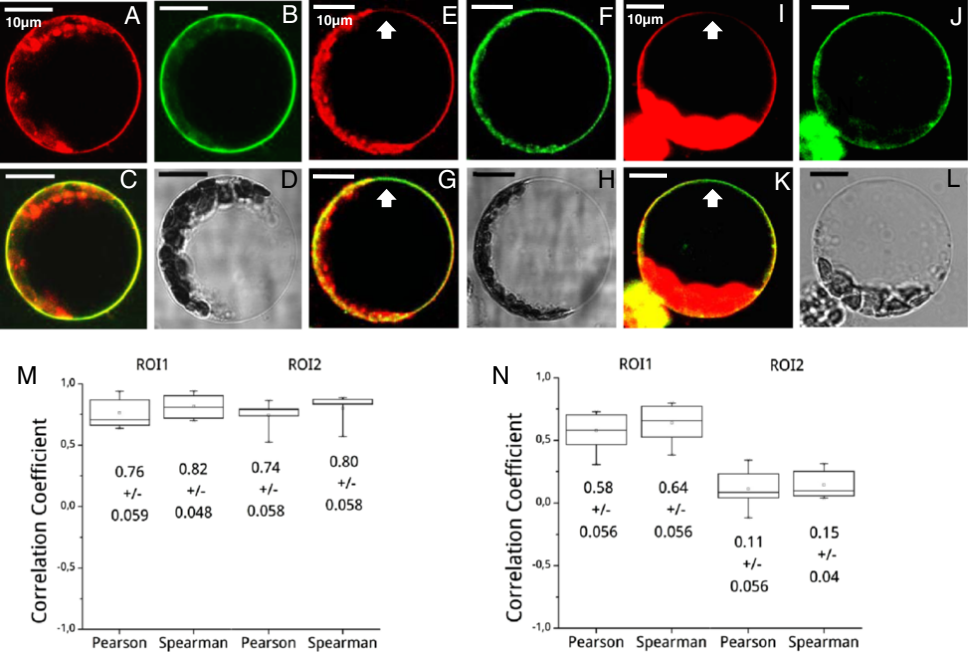
**Combined dye staining of protoplasts and correlation analyses.** There was no polarization detectable in freshly isolated, intact protoplasts. FM4-64 (**A**) & BD-SM (**B**) appeared to be homogeneous in the plasma membrane as viewed in the merged image (**C**) of the protoplast (transmission image (**D**)). (**E**-**F**)The occurrence of lipid polarization was observed 15 h post cell wall removal; (**E**) FM4-64 fluorescence, (**F**) BD-SM fluorescence; at the dorsal side there was a depletion of the FM4-64 fluorescence signal detected (**E**, **G** (merged image of **E** and **F**); arrows) in a viable protoplast (**H** (transmission image of the protoplast)). In 15 h old protoplasts these findings were confirmed by a combined use of LRB-PE (**I**) and BD-SM (**J**). A depletion of the LRB-PE fluorescence signal was also detected (**I**, **K** (merged image of **I** and **J**); arrows) in intact protoplasts (**L** (transmission image)). (**M**): Pearson and Spearman correlation coefficients resulting from two independent ROIs in freshly isolated, FM4-64/BD-SM treated protoplasts. Both coefficients indicated a colocalization of fluorescence signals. (n = 10 protoplasts). (**N**): In 15 h old protoplasts the determined correlation coefficients showed a decrease between unpolarized regions (ROI1) and polarized regions of the plasma membrane (ROI2). For ROI1 unpolarized regions were determined. ROI2 reflects the correlation of green BD-SM and red FM4-64 fluorescence signals next to ROI1 in unpolarized regions in (**M**) and in polarized regions (FM4-64 depleted) in (**N**) of the plasma membrane (n = 10 protoplasts). (See methods and Figure S2 for details of the analysis in **M** &**N**).

Based on experimental findings from experiments on model membranes and mammalian tissues we modified common staining protocols of the florescent probes FM4-64 (Figure 1, A-B; Figure 2, A-D), LRB-PE (Figure 2, E-F), DiIC_12_ (Figure 2, G-H), DiIC_18_ (Figure 2, I-J), DiD (Figure 2, K-L), BD-SM (Figure 1, C-D; Figure 3, A-B) and Laurdan (Figure 4) for their use in plants and documented their plasma membrane participation *in vivo*. Beyond the performance of single staining experiments of epidermal tissues (Figure 1), we optimized protocols for dual staining experiments on protoplasts (Figure 5). Dual staining was performed to create an assay by fluorescence microscopy to observe if other membrane constituents colocalize or segregate from sphingolipid-rich environments upon fluorescence labeling. The results presented here indicate that the dual staining separately labeled different lipid phases on individual cells. Hence we establish staining protocols suited for plant tissues and protoplasts, using commercially available fluorescent dyes, to not only describe the lipid segregation phenomenon in plants but also to establish a non-biochemical assay of protein and lipid colocalization. All staining protocols, dye-specific excitation/emission wavelengths and proper adjustments of the laser scanning microscopes are described in the methods section.

### Whole tissue staining

Using the standard manufacturer’s protocols, plant cells and tissues did not survive the staining procedures as observed by cell morphology and by trypan blue staining - presumably due to the high content of chloroform, alcohols and dimethylsulfoxide (DMSO). Thus, new strategies employing large dilutions of DMSO-stocks of florescent probes in aqueous solutions as well as the use of longer incubation times at room temperature (up to 4 h) and at 4°C (more than 4 h) displayed good staining results without causing any significant levels of cell death. Strong background fluorescence signals, especially for FM4-64 (Figure 1, A) and BD-SM (Figure 1, C), did not allow for proper imaging of individual cells. Instead fuzzy structures were seen which resulted from accumulations of the dyes within the microfibril textures of cell walls and from unspecific leaf autofluorescence signals (Figure 1). Some of the structures were of the plasma membrane, which we could verify by imaging with fluorescence protein-tagged membrane proteins in transgenic plant lines (data not shown), thus verifying previously recorded plant plasma membrane staining with FM4-64 [31]. To obtain good staining results cell wall components were enzymatically removed (see methods section).

### Single dye staining of protoplasts

The generation of protoplasts not only allowed better accessibility of the PM for the application of individual lipid analogues from the extracellular side but also helped to reduce unspecific fluorescence signals, which we mostly attribute to the unspecific staining of the cell wall and its components. The successful staining protocols were tailor-made for each lipid analogue dye. In each case, we verified that the plasma membrane was stained by comparison with the staining, imaging and colocalization of fluorescence protein-tagged membrane proteins in protoplasts from transgenic plant lines (data not shown).

### FM4-64

The lipophilic FM4-64 dye incorporates into the outer leaflet of plasma membranes where it is emitting an intense fluorescence between 580 nm and 650 nm. As soon as cell wall digestion was complete, the protoplasts were subsequently stained, using a final concentration of 0.5% (v/v) of the FM4-64 stock solution (1 μg/μl; see methods section). After an incubation period of 10-15 min at room temperature FM4-64 covered all areas of the plasma membrane homogenously (Figure 2, A). Twenty to sixty minutes after the initial staining period, fluorescent particles became visible inside the cytoplasm. Occurring endocytosis events became more enhanced with ongoing incubation periods (Figure 2, C). It has been reported that in tobacco BY2 cell suspension cultures as well as in BY2 protoplast suspensions (*Nicotiana tabacum* cultivar Bright Yellow 2 [32]) endocytosis events appeared 30 to 60 minutes after incubation with FM4-64. This is likely mediated by an active transport process since FM dyes cannot cross membranes due to their amphiphilic nature [33]. Ongoing endocytosis events at the plasma membrane indicated the protoplast’s viability, which was confirmed by trypan blue treatment (Figure 3, E). 30 minutes after incubation with FM4-64 an increasing number of fluorescent vesicles were detected inside the cytoplasm, indicating that endocytosis of the FM4-64 dye was still proceeding (Figure 2, C). FM4-64 is widely used as a non specific marker for endocytosis events and vesicle trafficking in living cells [34].

### LRB-PE

The head group labeled phospholipid Lissamine Rhodamine B-Phosphoethanolamine (LRB-PE) has its excitation maximum at 557 nm whereas the emission maximum is at 583 nm. In ternary lipid mixtures LRB-PE was found to favor L_d_-phases [35,36]. Cells were stained using 0.25% (v/v) of the LRB-PE stock solution dissolved in DMSO (1 μg/μl; see methods section). Following an incubation period of 20 minutes at room temperature cells were subsequently imaged using one-photon microscopy. Upon LRB-PE treatment the plasma membrane exhibited a homogeneous stain (Figure 2, E).

### DiIC’s and DiD

In this study two DiICs, DiIC_12_ and DiIC_18_, and DiD were tested. In model membranes DiIC_12_ and DiD partitioned into L_d_-phases, and DiIC_18_ partioned into both L_d_- and L_o_-phases [25,35,37]. Both DiICs were dissolved in DMSO (1 μg/μl). Protoplasts were stained using final concentrations of 0.5% (v/v) of DiIC_12_ and DiIC_18_ (see methods section). When used in protoplasts there were no preferred membrane regions for accumulation of either DiIC_12_ (Figure 2, G) or DiIC_18_ (Figure 2, I) molecules detectable. Both dyes augmented appeared in the cytoplasm within an hour, suggesting that DiIC_12_ as well as DiIC_18_ dyes underwent endocytosis events. In several staining experiments using different concentrations of DiD and expanding incubation times there were only weak fluorescence signals detectable. Again there was no plasma membrane compartmentalization observed using DiD (Figure 2, K).

### BD-SM FL C_12_ (BD-SM)

To counter-stain L_o_-phases a fluorescent sphingolipid analogue was employed, since i) lipids with mostly saturated hydrocarbon chains like sphingomyelins showed a preferred partitioning into L_o_-phases in model membranes [25] and ii) in DRM fractions obtained from plants sphingolipids were enriched [14]. For this reason the Bodipy Sphingomyelin FL C_12_ (BD-SM) was used to label L_o_-phases. According to information supplied by the manufacturer the BD-SM lipid analogue has the same stereochemical conformation as native, biologically active sphingolipids. Therefore BD-SM molecules incorporate into the plasma membrane and mix up with native sphingolipids. Phase partitioning experiments confirmed a participation of BD-SM in the DRM fraction (Figure 3, C). BD-SM was added right after the isolation and purification of Arabidopsis plasma membrane components. Following Triton X-100 treatment Bodipy fluorescence was detectable in the DRM-fraction, whereas FM4-64 and LRB-PE at the same time could not be detected using fluorescence microscopy (not shown). To label protoplasts a stock solution of 1 μg/μl BD-SM in DMSO was generated, using a final concentration of 1% (v/v) BD-SM/DMSO. In freshly isolated protoplasts BD-SM was homogenously distributed in the plasma membrane. The dye was hardly taken up into the cytoplasm within time periods of about 1 h; intracellular Bodipy fluorescence signals increased 15 to 18 hours (h) after labelling (Figure 3, A).

### Combined dye staining of protoplasts

The visualization of different lipid environments is clarified by the simultaneous staining of spectrally different fluorescent dyes with each color specifically attached to a liquid ordered or to a liquid disordered separating lipid. In the above experiments on Arabidopsis protoplasts, the best staining results were obtained using FM4-64, LRB-PE and BD-SM. DiD only weakly incorporated into plasma membranes, whereas DiIC_12_ and DiIC_18_ were strongly taken up into the cytoplasm, resulting in a decrease of fluorescence along time. To ensure solid fluorescence signals FM4-64 as well as LRB-PE were used individually to stain phospholipid enriched areas of the plasma membrane. BD-SM was employed at the same time to counter-stain sphingolipid enriched compartments.

In freshly isolated protoplasts there was initially a homogeneous stain of plasma membrane lipids detectable using combinations of FM4-64 and BD-SM (Figure 5, A-D). In the merge image a strong yellow color appeared for all areas of the plasma membrane, indicating a homogenous distribution of phospholipid- and sphingolipid-phases (Figure 5, C). Excitation of FM4-64 molecules with the 543 nm laser caused some cytosolic structures to show autofluorescence (Figure 5, A, E). As compared to the white light transmission image these structures were chloroplasts showing chlorophyll fluorescence at given wavelengths (Figure 5, D, H). Macroscopically the protoplasts appeared intact (Figure 5). 15 h after cell wall removal, the protoplasts underwent a rearrangement of lipids in the bilayer, resulting in a polarization of the plasma membrane. At the dorsal side a region appeared where a depletion of the FM4-64 signal was evident (arrows in Figure 5, E and G). The plasma membrane appeared no longer as homogenously stained (Figure 5, G). Polarization was even more enhanced 20 h after digestion of the cell wall (Additional file 1: Figure S1). In the protoplasts depicted FM4-64 fluorescence was strongly depleted at distinct sites of the membrane (Additional file 1: Figure S1, A-C; E-G; arrows). Time dependent lipid polarization was confirmed by a correlation analysis resulting from dye specific fluorescence signals (Figure 5, M and N). In freshly isolated protoplasts green BD-SM and red FM4-64 molecules appeared to be colocalized in the plasma membrane as indicated by high Pearson and Spearman correlation coefficients, ranging from 0.74 to 0.76 and 0.80 to 0.82 (Figure 5, M). With increasing time after enzymatically digesting the cell wall the correlation coefficients strongly decreased to 0.11 and 0.15 in polarized regions of interest (ROI’s) (Figure 5, N; for polarization analyses see also Additional file 2: Figure S2 and methods), which indicated an accumulation of lipids of a distinct phase. FM4-64 molecules were progressively detectable within the cytosol in time, indicating active endocytosis events at the plasma membrane to happen (Figure 2, A C). Since there are good hints that cellular endocytosis events depend on the presence of membrane sterols in plants [38], FM4-64 treatment might have induced changes in the lipid environment and thus the localization and activity of integral PM proteins. However, such changes were not observed in *A. thaliana* root, epidermis and cortex cells [31]. To reliably assure that FM4-64 uptake did not influence lipid polarization, experiments were repeated using LRB-PE. LRB-PE has been shown to be a stable plasma membrane marker that was hardly taken up into the cytosol (Figure 2, E). Again a lipid polarization was detected 15 h after cell wall removal as protoplasts were coevally stained using LRB-PE and BD-SM (Figure 5, I-K; arrows). A correlation analysis of LRB-PE and BD-SM fluorescence signals resulted in a dramatic change of the values of the correlation coefficients, decreasing from 0.89 in unpolarized protoplasts down to 0.39 in polarized ones. By the trend, these numbers strengthened the fact that plasma membrane resident lipids polarize in a time dependent manner. Nevertheless, we conclude that the time dependent differences in the plasma membrane lipid distribution can better be quantified by a FM4-64/BD-SM treatment rather than by a combined staining with LRB-PE and BD-SM.

### FRAP-experiments on polarized protoplasts

**Figure 6:**
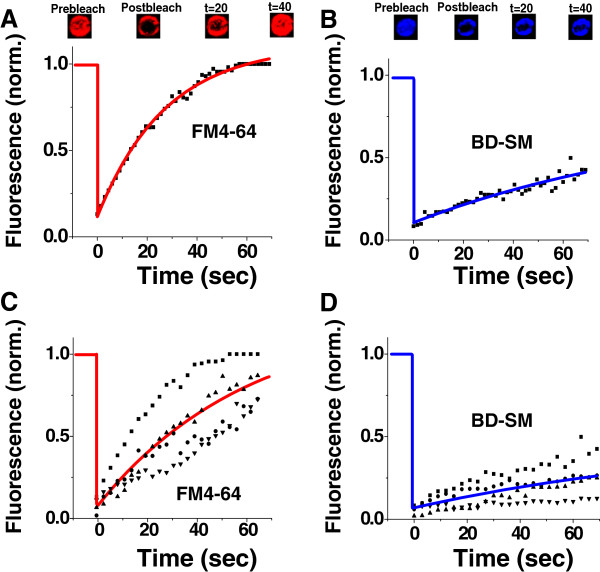
**FRAP-experiments on polarized protoplasts.** FRAP-experiments were performed on polarized areas of 15 h old protoplasts. (**A**) (Top) 10 μm x 10 μm image of FM4-64 in an FM4-64 enriched region before, right after, 20 s and 40s after the photobleach. (Bottom) Fluorescence recovery graph of FM4-64 from above protoplast region (data points as black squares) and with the recovery fitting (red line) depicting 100% recovery or complete mobility and a diffusion coefficient of 0.124 μm^2^/s. (**B**) (Top) 10 μm x 10 μm image of BD-SM in an FM4-64 depleted region before, right after, 20s and 40s after the photobleach. (Bottom) Fluorescence recovery graph of BD-SM from above protoplast region (data points as black squares) with a recovery fitting (blue line) depicting 48% recovery or a 48% mobile fraction and a diffusion coefficient of 3.4 × 10^-3^ μm^2^/s of the mobile fraction. (**C** &**D**) Four typical FRAP-measurements are depicted for each dye with the single data point measurements represented as black circles, squares and triangles; Fit of the average of the four typical curves for the manuscript reported values of the mobile fractionand diffusion coefficients for (**C**) FM4-64 in FM4-64 enriched regions and (**D**) BD-SM in FM4-64 depleted regions. (**C** – red curve &**D** - blue curve) (see manuscript). (See methods section for details of the FRAP acquisition and analysis.)

The usage of fluorescent dyes on *A. thaliana* protoplasts revealed time dependent lipid redistribution events to happen, resulting in a polarization of the plasma membrane. To verify a separation of different lipid species into liquid ordered/liquid disordered phases fluorescence recovery after photobleaching (FRAP) experiments were performed on polarized protoplasts to reveal if differences in these observed lipid domains might agree to mobility studies with different lipid composition. From unilamellar model membranes consisting of ternary mixtures of sphingomyelin, phosphatidylcholine (DOPC) and cholesterol, it was reported that the content of cholesterol determined lipid mobility in sphingomyelin enriched, liquid ordered phases. It was shown that an increase in cholesterol led to decreased lateral diffusion coefficients, likely as a consequence of lateral separation into a liquid ordered phase, enriched in cholesterol and sphingomyelin and a liquid disordered phase, predominantly containing DOPC [39]. Because of the dense packaging of sterols and sphingolipids it is assumed for *in vivo* systems that sterol-rich compartments reflect the features of liquid ordered domains in artificial membranes [26,40]. Based on these findings it was supposed that changes in the cholesterol content could be a possible tool for viable cells in tuning their membrane lipid dynamics [41]. In FRAP-studies on polarized protoplasts FM4-64 enriched and depleted regions were bleached out in the plane of the membrane (see methods for details). Protoplasts were successfully stained using final concentrations of 0.5% FM4-64 (v/v) and 1.0% BD-SM (v/v; see methods). In either case disc shaped regions with spot sizes of 1.5 μm in diameter were bleached (Figure 6). FRAP-studies demonstrated that areas with originally low or no FM4-64 but high BD-SM staining exhibit sedate recovery rates of BM-SM compared to the more accelerated ones of FM4-64 in FM4-64 enriched areas that also have approximately equal amounts of BD-SM. A diffusion coefficient [D] of 8.4 × 10^-3^ μm^2^/s and mobile fraction of 84% (or 16% immobility, n = 4) were observed and fitted in the FM4-64 enriched regions (Figure 6C). In contrast the mobility and mobile fraction were lower of the BD-SM in the areas with essentially no FM4-64 with a diffusion coefficient [D] of 8.01 × 10^-4^ μm^2^/s and mobile fraction of only 28% (72% immobility) (Figure 6, D n = 4). These numbers indicate that lipids in FM4-64 enriched environments have a higher percentage that are able to move much faster than lipids residing in FM4-64 depleted environments, which on average demonstrate little or no motion. FRAP-experiments emphasized the coexistence of at least two lipid phases in the plasma membranes of viable protoplasts.

### Laurdan

To verify the lipid polarization data, two-photon microscopy was employed using Laurdan as fluorescent probe. Laurdan is an environmentally sensitive dye that shows a 50 nm emission red-shift in its fluorescence spectrum in polar solvents and in the phospholipid liquid-disordered phase, as membranes undergo phase transitions from gel to fluid due to altered water contents in the lipid bilayer [42-44]. These differences in the plasma membrane water content can be visualized. Due to its molecular architecture Laurdans fluorescent naphthalene moiety exhibits a dipole moment between the 2-dimethylamino and the 6-carbonyl residues. Upon excitation the dipole moment increases; this increase is thought to cause a reorientation of surrounding solvent dipoles. The energy needed for the reorientation of surrounding solvents decreases the exited state energy of Laurdan and the emission spectrum of the probe is continuously red shifted when neighbored water molecules around reorganize [45]. Accordingly a red shift of the emission spectrum is observed in polar solvents, whereas in apolar solvents, such as in densely packed L_o_-phases with decreased water content, the emission is more blue-shifted. The false colored green images in Figure 4 reflect lipid ordered regions, whereas the red images reflect polar, lipid disordered sites of the plasma membrane with higher water content. To quantify specific shifts in the spectral behavior of Laurdan the generalized polarization (GP-) values were calculated [44,46]. The GP-value is dimensionless and reflects the predominant state of lipid order in lipid bilayers. In artificially generated ternary lipid mixtures liquid ordered domains have been characterized by high GP-values [46].

Protoplasts were stained immediately (Figure 4, A-E), 15 h (Figure 4, F-J) and 20 h (Figure 4, K-O) after an enzymatic digestion of cell wall components. Directly after cell wall removal the plasma membrane fluorescence was homogenous, which indicated that polar and apolar lipid regions were equally distributed throughout the whole plasma membrane at this point (Figure 4, A-D). A pixel to pixel calculation of the red and the green fluorescence signals revealed that there were no detectable differences in the lipid order, indicated by GP-values ranging from −0.3 to 0.2 (Figure 4, E). This situation changed with increasing age of the protoplasts. 15 h after cell wall removal a rearrangement of plasma membrane lipids was detectable (Figure 4, F-I). According to emitted Laurdan wavelengths the formation of two more blue shifted, apolar phases was observed (Figure 4, G; arrows). The apolarity content within these phases was confirmed by according GP-values ranging from 0.2 up to 0.6 - indicating sites of increased lipid order (Figure 4, J; arrows). 20 h after cell wall removal, polarization was even more enhanced (Figure 4, K-O; arrows). At the lateral side of the cell a highly ordered lipid phase emerged (arrow in Figure 4, O; GP-value between 0.6-0.8), whereas neighbored PM areas appeared to harbor less lipid ordered states (GP-values ranging from 0 to 0.2).

## Discussion

Here we present simple staining protocols suited to label intact plant tissues and protoplasts using fluorescent probes of FM4-64, LRB-PE, DiIC_12_, DiIC_18_, DiD, BD-SM and Laurdan. After initial incubations of Arabidopsis epidermal strips with either FM4-64 or BD-SM strong autofluorescence signals were detected that did not allow discriminations between dye-specific signals and unspecific ones coming from deeper leaf tissues (Figure 1). Some parts of the autofluorescence signal resulted from accumulations of the dyes within the cell wall microfibril texture. Therefore the plasma membrane participation of each dye in protoplasts was documented employing one- and two-photon microscopy. FM4-64 turned out to be a stable plasma membrane marker (Figure 2, C), just as LRB-PE (Figure 2, E) and BD-SM (Figure 3, A). DiIC_12_ (Figure 2, G) and DiIC_18_ (Figure 2, I) were strongly taken up into the cytosol which led to decreasing fluorescence signals at the plasma membrane over time. In contrast, a weak plasma membrane fluorescence was achieved using DiD (Figure 2, K). The dye hardly incorporated into the bilayer. To detect possible differences in the lipid composition of Arabidopsis, protoplast dual staining experiments were performed (Figure 5). There, we found evidence for lipid polarization in the plasma membrane. Plasma membrane lipids underwent redistribution events within 15 to 20 h after cell wall digestion. Using BD-SM as marker for sphingolipid enriched membrane compartments and other fluorescent markers like FM4-64 and LRB-PE for phospholipid enriched areas, lipid redistributions could be visualized (Figure 5, A-L). Lipid polarization was statistically allocated employing Pearson and Spearman correlation coefficients (Figure 5, M-N; [47]). In freshly isolated protoplasts BD-SM and FM4-64 showed colocalization in the plasma membrane, displayed by high correlation coefficients (Figure 5, M). Time dependent lipid polarization became evident by a strong decrease of the Pearson and Spearman correlation coefficients after 15 h (Figure 5, N), which indicated a more random distribution of dye molecules within the plane of the membrane. To ensure that the dyes labeled inhomogeneous lipid phases the partitioning of BD-SM in the Arabidopsis DRM fraction was confirmed by Triton X-100 treatment of purified Arabidopsis plasma membranes (Figure 3, C). Detergent treatment revealed that BD-SM mixed up with natural phytosterols and sphingolipids, which are thought to self-aggregate to form lipid clusters. In DRMs isolated from Arabidopsis callus cultures sterols and sphingolipids showed a 4- to 5-fold increase relative to the total plasma membrane [14,48]. In tobacco a similar increase of sterols and sphingolipids in DRMs was determined [49]. This indicated that BD-SM is likely labeling PM areas of elevated sterol/sphingolipid content *in vivo*. It was shown for sphingomyelins in model bilayer membranes that attached saturated acyl chains promoted the formation of raft-like lipid ordered phases that merged with each other to form larger domains [50].

Except for FM4-64 all dyes have been dissolved in dimethylsulfoxide (DMSO). DMSO/water or DMSO/buffer mixtures were used to stain Arabidopsis tissues and protoplasts. DMSO has been reported to have a strong influence on the structure of lipid membranes when used even at small mole concentrations, probably by displacing water and thereby modifying the structure of lipid bilayers [51]. Concerning cell biology, the amphiphilic DMSO molecule is known for its function to enhance membrane penetration, to induce cell fusion and for its role as cryoprotectant. Depending on DMSO concentration, membrane thickness can be affected, allowing for the formation of water pores to be induced and for the destruction of the bilayer structure of membranes [52]. In atomic-scale molecular dynamics simulations it was revealed for liquid disordered dipalmitoylphosphatidylcholine (DPPC/DMSO/water) systems that concentrations of 10-20 mol% of DMSO led to pore formations within timescales of nanoseconds [52]. Here we used DMSO concentrations in the range of 0.25-3% (v/v) which should not significantly alter the PM structure. Nevertheless there are reports using alfalfa protoplasts that a DMSO concentration of about 1% was already sufficient to induce changes in the transcript levels of certain genes, as reported for instance for cold acclimatization-specific (*cas*) genes. By increasing membrane rigidity, DMSO could possibly have induced calcium influxes, leading to a pronounced cold acclimation at room temperature [53]. Even so it cannot be ruled out that DMSO induced the expression of certain genes that could have influence on intracellular calcium concentrations as well as on the plasma membrane protein composition. In accompanied control experiments, however, there were no DMSO-induced effects on membrane polarization detectable when incubating Arabidopsis protoplasts for 24 h in buffer media containing up to 3% DMSO.

The FRAP-experiments left striking hints that there are at least two lipid populations resident in polarized poles of the plasma membrane. Lipid fractions that predominantly harbored fluorescent BD-SM molecules showed a significant decrease in recovery time compared to fractions in which FM4-64 was participating (Figure 6). The BD-SM labeled lipid fraction showed a diffusion coefficient [D] of 8.01 × 10^-4^ μm^2^/s with a mobile fraction of only 28% (a 72% immobile fraction), whereas for lipids in the FM4-64 labeled pool a [D] of 0.084 μm^2^/s with an 84% mobile fraction (a 16% immobile fraction) was determined. These numbers indicated that lipids of the FM4-64 labeled fraction are able to move more than 10 times faster than lipids of the mobile BD-SM labeled fraction, and the FM4-64 was predominantly mobile, whereas the BD-SM labeled fraction was predominantly immobile. In ternary systems consisting of cholesterol, sphingomyelin and dioleoylphosphatidylcholine (DOPC) diffusion coefficients were characterized with respct to lipid lateral diffusion rates. It was revealed that lipids of the L_o_-phase appeared to recover 2–3 times slower than lipids of the L_d_-phase [39]. In single gold particle tracking experiments on artificial giant unilamellar vesicles (GUV’s) an increase in the sterol lipid content up to 50% of all lipids in planar lipid bilayers led to a nearly two fold reduction of [D] [54]. At given temperature [D] strongly depends on the movement of surrounding lipids as well as from the observation period. In the fluid, lipid disordered phase of ternary model membranes large diffusion coefficients of about 10^-6^ cm^2^/s have been reported at short time scales of 1 ns, whereas at longer time scales diffusion coefficients decreased to 10^-8^ to 10^-7^ cm^2^/s [55,56]. With an assumed [D] of 10^-7^ cm^2^/s, an individual lipid would cover a lateral distance of about 6 nm within a time period of 1 μs. The applied FRAP-technique for the measurement of lateral diffusion rates of different lipid phases in viable protoplasts however did not allow time and length resolutions of this magnitude since this method is based on optical systems, whose spatial resolving power is limited by the chosen scan method and by the diffraction of light. Acquired FRAP datasets are, therefore, not directly comparable to diffusion coefficients calculated from atomistic simulations mimicking lipid diffusions in model membranes [55]. Nevertheless, there have been efforts made in defining lipid diffusion coefficients in model membranes employing FRAP; in pure liquid disordered, dimyristoylphosphatidylcholine (DMPC) systems a [D] of 7.5x10^-8^ cm^2^/s was measured at 35°C, decreasing to 6.0× 10^-8^ cm^2^/s when temperature dropped to 26°C. As cholesterol was added to the same system the liquid ordered phase formed; at 35°C [D] was measured to be 3.0×10^-8^ cm^2^/s in the L_o_-phase, being further reduced to 1.8×10^-8^ cm^2^/s at 26°C [57]. FRAP measurements in protoplasts were carried out at room temperatures of about 20-22°C. Taking the pure numbers, lipid diffusion coefficients in protoplasts appear to be two to three orders of magnitude slower compared to those found in artificial membranes.

To our best knowledge no similar FRAP-datasets are available describing lateral lipid diffusion coefficients in viable Arabidopsis protoplasts to this date. In analogy to findings in model membranes it is assumable for polarized protoplasts that the slower fluorescence recovery rates of BD-SM phases are caused by an accumulation of sterols at distinct sites of the plasma membrane (Figure 5, G, K; Figure 6).

Different dye loadings and FRAP-experiments revealed plasma membrane lipid heterogeneity in Arabidopsis protoplasts. One further possibility to prove this finding was the employment of a lipid environment sensitive fluorescent probe like Laurdan. Since in L_o_-phases sterols and sphingolipids are tightly packed these regions have a less water content compared to L_d_-phases, in which lipids are more densely packed. Laurdan can be applied to visualize such differences in the water content. In Experiments on *A. thaliana* protoplasts, a final Laurdan concentration of 5 mM was enough to successfully stain viable plasma membranes. For mammalian cells in contrast concentrations in the micromolar range are used. This might be explained by the more complex sterol profile of plant plasma membranes.

In L_o_-phases fluorescence is shifted into the more blue spectrum of light (false colored green in Figure 4), in polar L_d_-phases accordingly more into the red. Immediately after cell wall removal there were no lipid phase polarizations detectable (Figure 4, A-C), confirmed by a calculation of the general polarization (GP-) value in a pixel to pixel analysis of Laurdan images (Figure 4, E). The GP-value can be used as indicator for the water content of lipid phases and accordingly as a degree for the predominant state of lipid order [43]. A GP-value of −1 indicates an aqueous phase, whereas a GP-value of +1 indicates a fully ordered phase. GP-values in Figure 4 E ranged from −0.3 to 0.2. Experimentally these values depend on the lipid composition and on temperature. In the liquid disordered phase of model membranes GP-values ranged from −0.3 to 0.3 while in liquid ordered phases these values are typically in the range of 0.5 to 0.6 [45]. In liposomes with equal molecular ratios of DOPC, cholesterol and sphingomyelin the GP-values of the liquid disordered phase ranged from −0.05 to 0.25 whereas a GP of 0.25 to 0.55 indicated the coexistence of a liquid ordered phase [42]. It has been reported that “GP-values of living cells may not always directly correspond to those obtained in artificial membrane systems”, but that GP-values allow comparisons in order to rule out fluidity differences within plasma membranes [42]. Recently Laurdan has been used to examine the degree of lipid order in different tissues of vertebrates. In vital zebrafish embryos the quantification of membrane order ranged between GP-values of 0 to 0.2 in more ordered apical membranes compared to basolateral membranes exhibiting a decreased lipid order with GP-values of 0 to 0.13 [58]. In mammalian MDCKII and RAW264.7 cell lines GP-values ranged from −0.1 to 0.3. Using the sterol-depleting agent methyl-ß-cyclodextrin GP-values decreased with decreasing sterol content of the plasma membrane, examined by a fluorescent live imaging approach [59]. Using Laurdan on native BY2 plasma membranes GP-values dropped from 0.65 (at 4°C) to 0.55 (at 22°C) down to 0.30 (at 40°C). In analogy to findings in model membranes the BY2 plasma membrane constitution would accordingly be consisting of gel-like, liquid ordered domains below 20°C (GP > 0.55); above this temperature the relative order of the membrane progressively decreased [60].

Laurdan treatments of Arabidopsis protoplasts revealed that 15 h after cell wall removal redistributions of lipid phases occur (Figure 4, F-H). With respect to the Laurdan fluorescence characteristics there were lipid phases with different water content emerging (Figure 4, F, G). GP-values of up to 0.6 indicated high levels of lipid order for regions of the plasma membrane (Figure 4 J, arrows). After 20 h polarization was even more pronounced, resulting in one distinct lipid ordered pole covering the lateral side of the protoplast (Figure 4, K-O, arrows). Likely this pole arose from the coalescence of smaller regions of lipid order, as seen in Figure 4, J. For model membranes it has been reported that L_o_-phases, predominantly harboring lipids with saturated acyl chains like sterols and sphingomyelins, merged with each other to form larger domains [50].

Lipid polarization could be confirmed by several independent non-invasive tests like staining experiments with different lipid analogues (Figure 5 A-L), FRAP-measurements (Figure 6) and Laurdan labeling of plasma membranes including a calculation of GP-values (Figure 4). At this point it is unclear what exactly caused plasma membrane resident lipids to relocate into distinct poles. Polarizations of lipid domains are reported for several biological systems across species, especially during cytokinesis [61]. Experiments in fission yeast (*Schizosaccharomyces pombe*) revealed that there are sterols enriched at the growing cell tips and at sites of cytokinesis [62]. In pollen tubes lipid microdomain polarization was found to be essential for polarized tube growth involving reactive oxygen species (ROS) signaling. ROS-producing NADPH oxidases were reported to be associated with DRMs and to depend on the presence of sterols [63]. ROS signaling plays important roles in plants since reactive oxygen species help controlling processes of growth, development, biotic and abiotic stress response and programmed cell death [64]. In the experiments performed here there were in contrast neither cell divisions nor any kinds of developmental processes detectable during observation periods of up to 24 h.

It has also previously been reported that plant protoplasts start regenerating their cell walls in suited cultivation media. In *Nicotiana tabacum* protoplast cell wall regeneration started within 30 minutes after removing cell wall components, and cell wall regeneration processes were indicated by formations of cellulose microfibril depositions at distinct sites at the protoplasts surface [65]. The spatial organization of cellulose microfibrils defines the cell wall texture and is achieved by an oriented microfibril deposition. Transmembrane cellulose-synthase-complexes are directly linked to components of the cytoskeleton and enable an organized deposition of cellulose microfibrils [66]. Some of the cell wall building components like chitin- and ß-D-glycan-synthases have been shown to be resident in detergent resistant membrane domains of Oomycetes [67]. Callose and cellulose synthases have also found to be strongly enriched in DRM fractions isolated from plasma membranes of the tree species Populus, indicating that active proteins involved in cell wall biosynthesis associate with sphingolipid/sterol enriched, lipid ordered phases *in vivo*[68]. It is likely that lipid redistributions in plasma membranes of Arabidopsis protoplasts reflect cellular attempts to regenerate absent cell walls. In tobacco protoplast cell walls fully regenerated within a time period of up to six hours. Observations were made, that the period in which the regeneration processes finished, strongly depended on the method that was used before when isolating the protoplasts; depending on the isolation procedure the regeneration processes could take up to 16 hours [65]. Nevertheless there were no indicators for cell wall regenerations found in Arabidopsis protoplasts during observation periods of up to 24 h. This could be due to the medium used for cultivating the protoplasts; the medium still contained intact proteolytic enzymes like cellulases and pectolyases, which could have hindered cell wall regenerations. In *Convolvus arvensis* protoplasts it was shown that the ability of single cells to regenerate their cell walls was strongly diminished in the presence of active proteolytic enzymes [69].

## Conclusions

The basic knowledge in terms of lipid behavior derived from experiments in artificial membranes of defined lipid mixture. In viable cell membranes this knowledge is limited, since there are still missing links concerning lipid behavior and lipid composition. Lipophilic fluorescent dyes and lipid analogues could help broadening our knowledge from model membranes to functional ones. So far a majority of the emerging bulk of commercial available dyes and lipids suited for fluorescent real time imaging is designed to stain mammalian cells and is - because of the altered lipid composition - not suited for the use in plants. Designing dyes and lipids basically for the usage in mammalian cells surely is owed by the fact that plant lipidomics have so far been of limited interest only. Within the last years there are trends indicating that this situation is changing. Here we took attempts to adapt some of the mammalian staining protocols to viable plant cells and documented the plasma membrane localization of FM4-64, LRB-PE, DiIC_12_, DiIC_18_, DiD, BD-SM and Laurdan in Arabidopsis protoplasts. Optimizing the staining protocols we found evidence that there are different lipid phases emerging in the plasma membranes of protoplasts 15-20 h after an enzymatic digestion of the cell wall. Dual labeling, as well as, FRAP experiments in polarized plasma membrane areas and Laurdan based GP-value calculations confirmed this assumption. The most plausible explanation seems that these lipid polarizations are reflecting cellular efforts to restore the cell walls of protoplasts. Nevertheless more experiments are needed to discover the molecular origin of this redistribution of lipids. Plant-adapted staining protocols included here might alleviate future approaches to investigate lipid compositions in viable plant cells. The use of fluorescent dyes and lipid analogues suited for confocal laser scanning microscopy and related techniques enables the study of lipid dynamics and lipid distributions in viable cells in real time.

## Methods

### One and two photon microscopy

Images were obtained using confocal microscopes (Zeiss LSM5 Pascal; Carl Zeiss Microimaging, Jena, Germany or TCS SP5; Leica, Mannheim, Germany). For two-photon microscopy approaches a femtosecond pulsed Ti:Sa-laser (Mai Tai, Spectra Physics; Darmstadt, Germany) was utilized, coupled into a TCS SP5 system (Leica) (Table 1).

**Table 1:** LSM-Filterset/TCS SP5 Emission settings

	**Exc. (nm)**	**Emm. (nm)**	**Emm. max. (nm)**
FM4-64	543	580-650	640
LRB-PE	543	580-630	583
DiICs	543	560-620	565
DiD	543	640-680	665
BD-SM	488	500-550	520

### Whole tissue staining

Epidermis strips of adult *A. thaliana* leaves were stripped off of the whole leaf and kept in water. The utilization of epidermal strips helped reducing background autofluorescence signals and further enabled a full tissue penetration by employed laser beams. For proper staining experiments dyes were either solved water (FM4-64; 1 μg/μl) or in dimethylsulfoxide (BD-SM; 1 μg/μl). Based on the stocks individual aqueous staining solutions were prepared. Arabidopsis epidermal strips were incubated for 15 minutes with FM4-64 at a final concentration of 1.0% (v/v) or with BD-SM (up to 30 minutes at a final concentration of 5.0% v/v). Directly after staining the strips were shortly incubated in pure water to wash away excessive dye molecules.

### Single dye staining of protoplasts

#### FM4-64

{N-(3-triethylammoniumpropyl)-4-(6-(4-(diethylamino)phenyl)hexatrienyl)pyridium dibroide}

The lipophilic FM4-64 dye is based on a polyethylene structure. The dye is water soluble and non toxic for living cell tissues according to manufacturers’ information (Invitrogen; Karlsruhe, Germany); the viability of the cells was additionally confirmed by trypan blue treatment (Figure 3, E). The dye was solved in pure water to a stock solution of 1 μg/μl. In staining experiments a final concentration of 0.5% (v/v) was used to stain Arabidopsis protoplasts. After an incubation period of 10 to 15 minutes at room temperature in buffer medium cells were fairly stained. A wavelength of 543 nm was employed to excite the fluorophore; the emission spectrum of FM4-64 was in the range of 580-650 nm (emission max.: 640 nm).

### Lissamine rhodamine B-phosphoethanolamine (LRB-PE)

{1,2-dimyristoyl-*sn*-glycero-3-phosphoethanolamine-N-(lissaminerhodamine B sulfonyl)}

LRB-PE (Avanti Polar Lipids; Alabaster, USA) was solved in dimethylsulfoxide (DMSO) to a stock concentration of 1 μg/μl. Protoplasts were incubated for 20 minutes at room temperature at a final concentration of 0.25% (v/v) LRB-PE/DMSO in protoplast buffer. An excitation wavelength of 543 nm was used to excite the fluorophore; emitted light was collected between 580 and 630 nm (emission max.: 583).

### DiIC_12_ & DiIC_18_

{DiIC_12_: (1,1^′^-didodecyl-3,3,3^′^,3^′^-tetramethyl-indocarbo-cyanine perchlorate)}

{DiIC_18_:(1,1^′^-dioctadecyl-3,3,3^′^,3^′^-tetramethyl-indocarbocyanine perchlorate)}

Both lipophilic DiIC dyes (Invitrogen) were solved in DMSO to a stock concentration of 1 μg/μl. Protoplasts were incubated using a final concentration of 0.5% (v/v) DiIC_12_/DMSO or rather DiIC_18_/DMSO in protoplast buffer. After an incubation time of 20 minutes at room temperature protoplasts were scanned with the laser scanning microscopes, employing a wavelength of 543 nm to excite the fluorophores; emission was collected between 560 and 620 nm (emission max: 565).

### DiD

{(1,1^′^-dioctadecyl-3,3,3^′^,3^′^-tetramethylindodicarbocyanine 4-chlorobenzenesulfonate salt)}

For the application of the lipophilic DiD dye (Invitrogen) a stock concentration of 1 μg/μl in DMSO was generated. To label protoplast suspensions various concentrations were generated, ranging between 0.25 and 3.0% DiD/DMSO (v/v). Using different DiD concentrations only weak fluorescence signals were detectable at the plasma membrane (along with increased incubation periods of up to 30 minutes). DiD was excited using a 543 nm laser, showing its maximal emission at 665 nm.

### Bodipy-sphingomyelin FL C_12_ (BD-SM)

{N-(4,4-difluoro-5,7-dimethyl-4-bora-3a,4a-diaza-*s*-indacene-3-dodecanoyl)sphingosyl phosphocholine)}

The amphiphilic BD-SM lipid analogue consists of a sphingosine moiety which is linked to a fatty acid by an amide bond. The sphingomyelin itself is covalently linked to a Bodipy fluorophor (Invitrogen). Referring to information of the manufacturer BD-SM features the same stereochemical conformation than biologically active sphingomyelins, in spite of the fluorescence labeling. The bodipy fluorophore exhibits similar emission and excitation wavelengths like the green fluorescent protein, GFP (exc.: 488 nm, em. 500-550 nm). The dye was solved in DMSO to a stock concentration of 1.0 μg/μl. Protoplasts were stained using 1% (v/v) of the stock solution in protoplast buffer. After an incubation time of 20 minutes at room temperature cells were fairly stained.

### Combined dye staining of protoplasts

To label putative different lipid phases, protoplast suspensions were co-incubated using stocks of FM4-64, LRB-PE and BD-SM (all solved in DMSO to 1 μg/μl). In dual labelling experiments the protoplasts were coevally incubated using 0.5% FM4-64 (v/v) and 1% BD-SM (v/v) or LRB-PE (0.25% v/v) and BD-SM (1% v/v). Protoplasts were incubated for 20 minutes at room temperature and subsequently imaged.

### Correlation analysis

Correlation analyses were performed using the colocalization plug-in for Image J (v. 137.c), as developed by French *et al.*[47]. The plug-in allowed a performance of quantitative statistical colocalization on two-color confocal images. For each protoplast two regions of interest (ROI) were chosen and the Pearson and Spearman correlation coefficients calculated (an example of a correlation analysis is given in Additional file 2: Figure S2). For polarized protoplasts one ROI (ROI1) was unpolarized or was enriched with both lipid-dyes and the other region (ROI2) was the predominately depleted FM4-64 region. If the items are correlated, they will have a value >  0.1 to 1. If the colors are uncorrelated (meaning separated), they will have a value of < −0.1 to −1. If the colors are randomly correlated or if only one color appears within a region, the value is essentially zero or approximately > −0.1 and <0.1. The Spearman coefficient was calculated in addition to the one of Pearson, since it does not assume the linear relationship of statistic variables. The usage of both coefficients to determine possible correlations allowed for higher statistic significance. All correlation coefficients are displayed as box-charts, each one showing the highest/lowest individual correlation value, the standard error bar for the values displayed, the average correlation value and the standard deviation for each ROI.

### Laurdan

{6-lauroyl-2-dimethylaminonaphthalene)}

Laurdan was dissolved to 60 mM in a solution of DMSO containing 17% ethanol (| > 97%). The final concentration in a distinct protoplast suspension was 5 mM; after incubation times of up to 60 minutes at room temperature cells were sufficiently stained. To generate desired excitation wavelengths of about 390 nm a multiphoton (MP-) laser at 760 nm (Mai Tai, Spectra Physics) was employed. Blue light was gathered between 400 to 440 nm (Intensity blue); the red shifted spectrum was collected using a bandpass ranging from 490 to 550 nm (Intensity red).

### Pixel by pixel analysis

To quantify changes in the Laurdan emission spectra pixel to pixel analyses of the blue and the red signals were performed. The predominant lipid order in different areas of the plasma membrane was measured as “*generalized polarisation*” (or GP-) value, following the equation

$$(I_{b})-(I_{r})/(I_{b})+(I_{r})=\text{GP}\text{.}$$

I_b_ and I_r_ correspond to the intensities of the blue and the red emission spectrum [46]. Liquid ordered regions show high GP-values since these regions are enriched in sterols. An enrichment in sterols lead to poor water levels. The membrane gets apolar and Laurdan emission and excitation wavelengths are more shifted into the blue spectrum. The GP-value is dimensionless, ranging from −1 (less ordered phase, polar lipid surrounding) to +1 (fully ordered phase, apolar lipid surrounding).

### *Arabidopsis thaliana* protoplast isolation

Leaves of 8 to 10 week old *A. thaliana col 0* plants were cut into small sections (1×1 cm) and were incubated for 2 h in digestion buffer at 28°C in an incubation shaker (30 rpm). The digestion buffer contained cellulase (0.8% w/v; Onozuka R10; Onozuka, Yakult Pharmaceutical Industry; Tokyo, Japan), pectolyase (0.1% w/v; Sigma Aldrich; Taufkirchen, Germany), bovine serum albumin (0.5% w/v) and polyvinylpyrolidone (0.5% w/v); finally calcium chloride was added (1 mM). The osmolarity was adjusted to 280 milliosmol/kg using sorbitol; the pH was adjusted to 5.6 (MES-Tris). After incubation a short centrifugation step was necessary (80-100 rpm, 10 min, 4°C) to separate undigested leaf fragments from the protoplasts.

### Cell viability

Trypan blue stain (0.4%; Lonza; Walkersville, USA) was used as an indicator for cell viability. Trypan blue was solved to 1 mg/ml in 0.6 M mannitol. Nonviable cells absorb the dye and appear blue while intact cells and membranes remain unstained. The dye was directly applied into the protoplast buffer, and the suspension was carefully mixed. To obtain a good black-white contrast suited for imaging Trypan blue concentrations of up to 20% (v/v) were used. After 10 minutes incubation at room temperature cells were sufficiently stained.

### DRM-isolation

Detergent-resistant membranes from plasma membranes were generated from *Arabidopsis thaliana* leaves as described elsewhere [49,70,71]. Before the extraction of the DRM-fraction by using 1% v/v Triton X-100, BD-SM (1.0 μg/μl) was added to the purified plasma membrane. BD-SM and plasma membrane lipids were mixed until the suspension cleared up. Subsequently 1% Triton X-100 was applied (optimized as determined in [49]), followed by a 16 h sucrose density centrifugation. The DRM-fraction was extracted and aliquots were used for confocal microscopy studies.

### FRAP acquisition and analysis

The FRAP-data were acquired with either the Leica LASAF FRAP Wizard or Zeiss LSM5 Pascal FRAP program, under the imaging conditions listed above. The bleach area on the surface of the plasma membrane was always a 1.5 μm diameter disc. The data could be acquired every 0.25 seconds but were reduced to 1.5 seconds between analysis points for the purposes of this manuscript. The images were analyzed with Image J (NIH, USA) and the data was exported to Origin (Origin Labs, USA). Average fluorescence intensities within ROIs in the bleached regions were analyzed for each detection channel to obtain the recovery data. Corrections were made for photobleaching during scanning by monitoring neighboring cells and analyzing their signals. Mobile fractions were calculated and the recovery was fit to the equation: I = A1 - A2*exp(−k*t) where I is the average intensity, k is the rate of the exponential recovery, t is the time, A1 is the full recovery value and A2 is the value of the drop in intensity after the bleach. The lateral diffusion coefficients, D, were determined from $D=\left(\frac{\beta \omega^{2}}{4ln2}\right)k$, where β is the bleach depth (determined to be 1.2), ω is the radius of the bleach area and k is the fitted rate constant.

## Competing interests

The authors declare that they have no competing interests.

## Authors’ contributions

JOB developed new and modified existing protocols suited for staining viable plant tissues and protoplasts; performed SP- & MP-microscopy; analyzed acquired digital images; interpreted the data and drafted the manuscript. FD isolated Arabidopsis DRM-fractions and provided support during staining experiments. IF helped coordinating the experiments and gave useful advice. GSH conceived of the study and its design; drafted parts of the manuscript and was an advisory capacity in terms of confocal imaging. RH assisted in conceiving the study and provided advice regarding the results. GSH, FD & IK helped correcting the manuscript, which final version all authors have read and approved.

## Supplementary Material

Additional file 1 **Figure S1.** FM4-64/BD-SM staining of protoplasts. FM4-64/BD-SM staining on protoplasts, 20 h post cell wall removal. Polarization was even more enhanced after this time period (A-H). (A; E) FM4-64 fluorescence. (B; F) BD-SM fluorescence. In the merged images clear-cut polarizations were detected (C; G, arrows). Tranmission images (D; H). Click here for file

Additional file 2 **Figure S2.** Example for correlation analyses. For statistically relevant correlation analyses two ROI’s within the plasma membranes of protoplasts were chosen and the Pearson and Spearman correlation coefficients calculated from confocal images (see methods section). The images selected are of (A) an unpolarized protoplast from Figure 5, C and two 15 h old protoplasts (B) one with FM4-64 staining from Figure 5, G and (C) one with LRB staining from Figure 5, K. (A – C) ROI1 was placed into non-polarized membrane areas and served as negative control for putative correlation. (A) ROI2 reflects the correlation of green BD-SM and red FM4-64 fluorescence signals next to ROI1 in unpolarized regions in unpolarized protoplasts. (B & C) In 15 h old protoplasts ROI2 was exclusively placed to ROI1 neighboring polarized areas. High correlation coefficients indicated a colocalization of pixels of the two different colors (green and red), whereas negative correlation coefficients indicated a separation of the two colors. Click here for file
