# Supplementary figures and images for: Methods of staining and visualization of sphingolipid enriched and non-enriched plasma membrane regions of Arabidopsis thaliana with fluorescent dyes and lipid analogues
Source: Plant Methods. 2012 Aug 6;8:28. doi: 10.1186/1746-4811-8-28 (PMC3544639; doi:10.1186/1746-4811-8-28)

## Slide 1
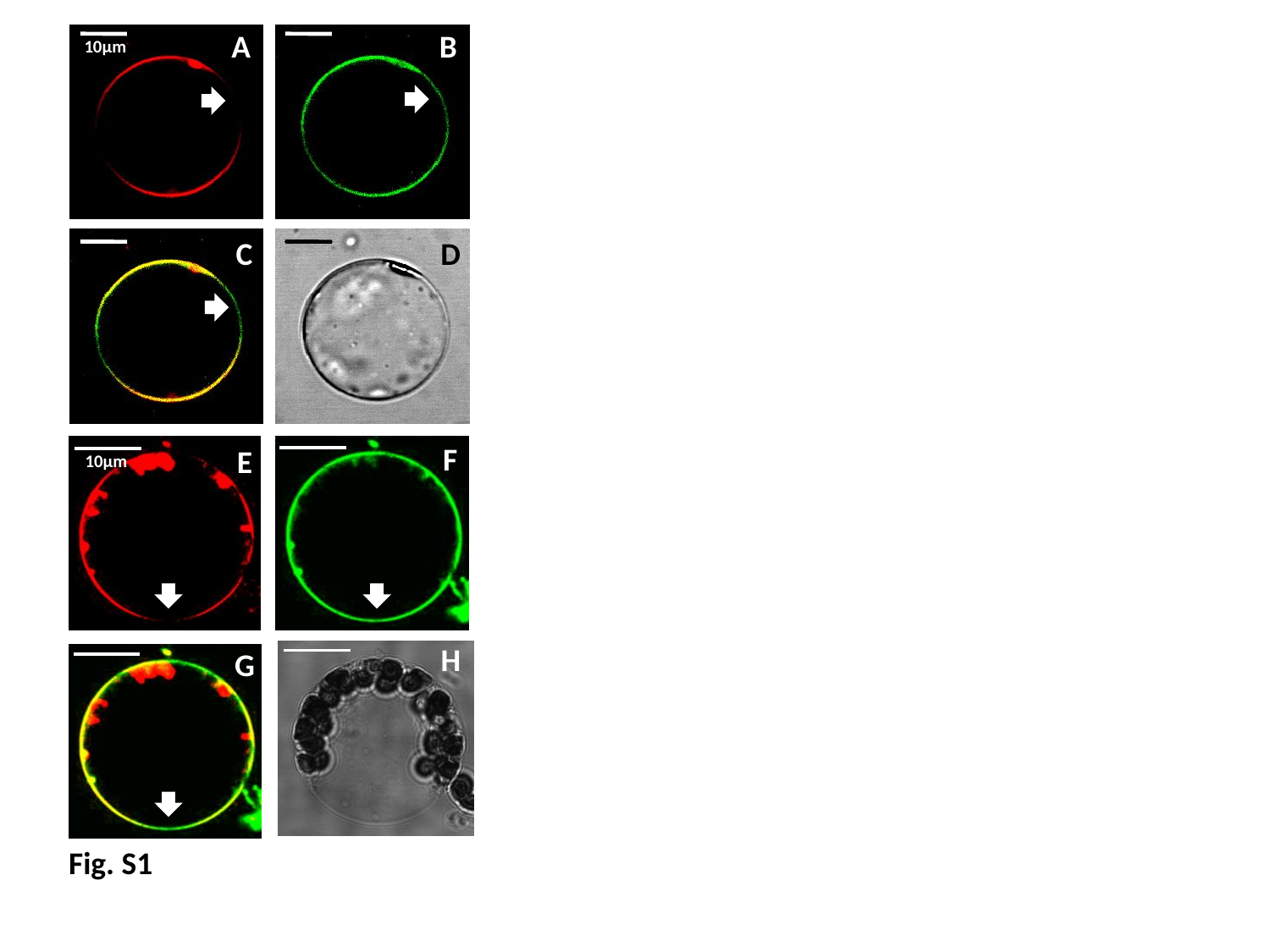

A
B
10
µ
m
10
µ
m
A
B
10
µ
m
C
D
10µm
C
D
F
E
10µm
H
G
Fig. S1

Supplement: Additional file 1 — Figure S1. FM4-64/BD-SM staining of protoplasts. FM4-64/BD-SM staining on protoplasts, 20 h post cell wall removal. Polarization was even more enhanced after this time period (A-H). (A; E) FM4-64 fluorescence. (B; F) BD-SM fluorescence. In the merged images clear-cut polarizations were detected (C; G, arrows). Tranmission images (D; H). [file 1746-4811-8-28-S1.ppt]
