# Supplementary material for: Methods of staining and visualization of sphingolipid enriched and non-enriched plasma membrane regions of Arabidopsis thaliana with fluorescent dyes and lipid analogues
Source: Plant Methods. 2012 Aug 6;8:28. doi: 10.1186/1746-4811-8-28 (PMC3544639; doi:10.1186/1746-4811-8-28)
Supplement: Additional file 2 — Figure S2. Example for correlation analyses. For statistically relevant correlation analyses two ROI’s within the plasma membranes of protoplasts were chosen and the Pearson and Spearman correlation coefficients calculated from confocal images (see methods section). The images selected are of (A) an unpolarized protoplast from Figure 5, C and two 15 h old protoplasts (B) one with FM4-64 staining from Figure 5, G and (C) one with LRB staining from Figure 5, K. (A – C) ROI1 was placed into non-polarized membrane areas and served as negative control for putative correlation. (A) ROI2 reflects the correlation of green BD-SM and red FM4-64 fluorescence signals next to ROI1 in unpolarized regions in unpolarized protoplasts. (B & C) In 15 h old protoplasts ROI2 was exclusively placed to ROI1 neighboring polarized areas. High correlation coefficients indicated a colocalization of pixels of the two different colors (green and red), whereas negative correlation coefficients indicated a separation of the two colors. [file 1746-4811-8-28-S2.ppt]

## Slide 1
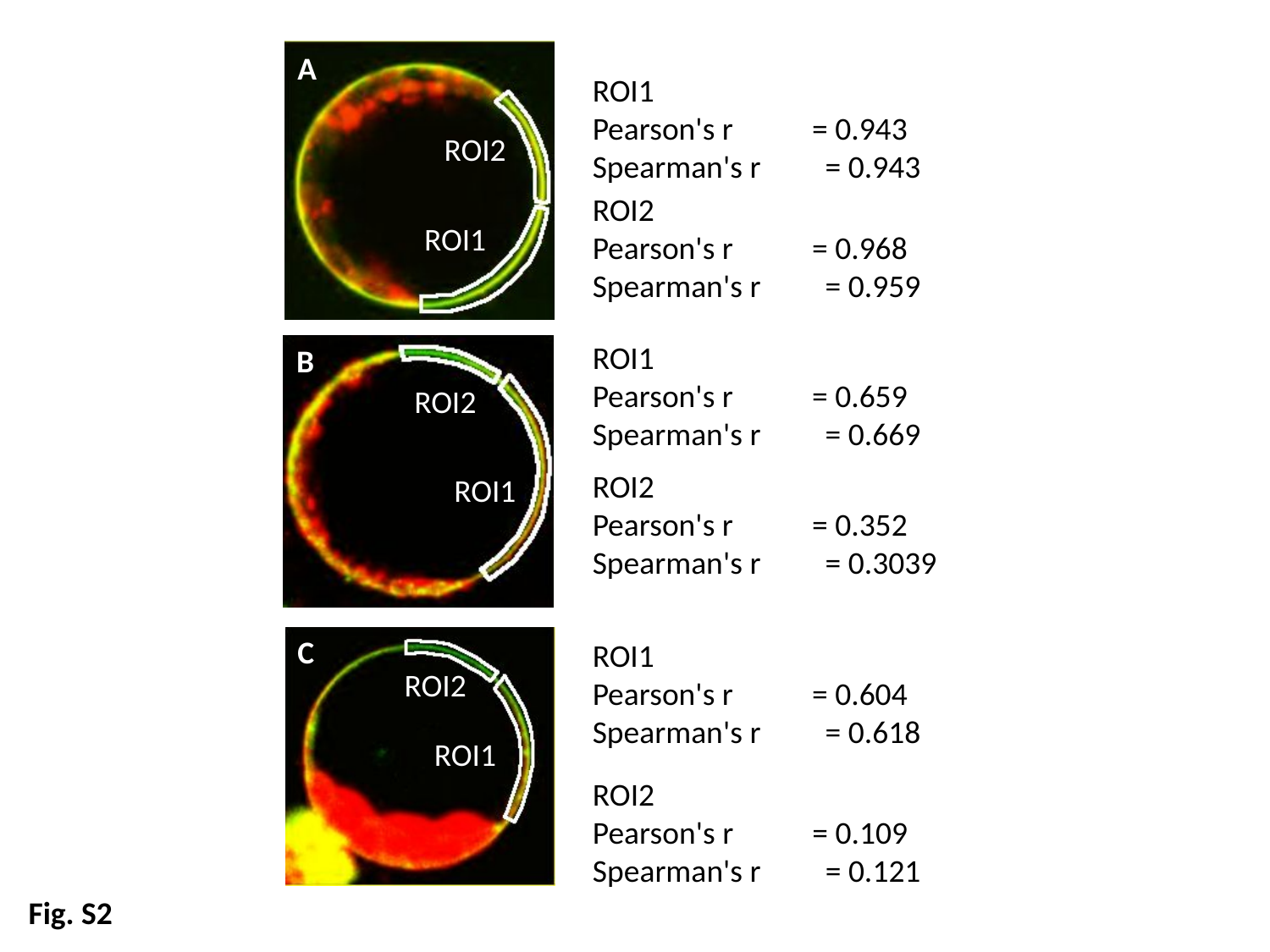

ROI2
ROI1
A
ROI1
Pearson's r = 0.943
Spearman's r = 0.943
ROI2
Pearson's r = 0.968
Spearman's r = 0.959
ROI1
Pearson's r = 0.659
Spearman's r = 0.669
B
ROI2
ROI1
ROI2
Pearson's r = 0.352
Spearman's r = 0.3039
C
ROI1
Pearson's r = 0.604
Spearman's r = 0.618
ROI2
ROI1
ROI2
Pearson's r = 0.109
Spearman's r = 0.121
Fig. S2
